# Long-Term Risk of Stroke After Snake Envenomation: A Nationwide Population-Based Cohort Study in Korea

**DOI:** 10.3390/toxins18060265

**Published:** 2026-06-12

**Authors:** JeongMi Moon, ByeongJo Chun, EuJene Jung, DongKi Kim, YeonJi Seong

**Affiliations:** 1 Department of Emergency Medicine, Chonnam National University Hospital, Gwangju 61487, Republic of Korea; 2 Department of Emergency Medicine, Chonnam National University Medical School, Hak Dong 8, Gwangju 61469, Republic of Korea; 3 Department of Emergency Medicine, Chonnam National University Hwasun Hospital, Hwasun 58128, Republic of Korea

**Keywords:** cohort study, hemorrhagic stroke, snake bite, snake envenomation

## Abstract

Snake envenomation causes acute cerebrovascular complications, but its long-term effect on stroke risk remains unclear. This study suggests that snake envenomation may be associated with long-term stroke risk. Using the Korean National Health Insurance Service database, we conducted a nationwide population-based cohort study to evaluate the long-term risk of stroke following snake envenomation. A total of 764 adult patients diagnosed with snake envenomation and treated with antivenom were identified and matched with 3056 control patients (1:4) by age, sex, and socioeconomic status, excluding those with prior cerebrovascular disease. Stroke outcomes were defined using ICD-10 diagnostic codes and healthcare utilization criteria. After a 1-year lag period was applied to minimize reverse causation, multivariable Cox proportional hazards models were used to estimate adjusted hazard ratios for total, ischemic, and hemorrhagic strokes. During 10 years of follow-up, snake envenomation was associated with a significantly increased risk of total stroke (aHR 1.42 (95% CI 1.01–1.99)), particularly hemorrhagic stroke (aHR 2.55 (95% CI 1.12–5.80)), whereas no significant association was observed with ischemic stroke. Interaction analyses showed a stronger association among men with diabetes mellitus, particularly for hemorrhagic stroke. In addition, severe envenomation with disseminated intravascular coagulation or requiring transfusion was associated with a higher long-term risk of hemorrhagic stroke. These findings highlight the need for further investigations of long-term cerebrovascular complications of snake envenomation, particularly hemorrhagic stroke in vulnerable populations.

## 1. Introduction

In 2009, World Health Organization designated snakebite envenoming as a neglected tropical disease. The Viperidae and Elapidae families constitute the major venomous snakes worldwide [1,2,3]. In South Korea, the terrestrial venomous snakes include three *Gloydius* species (*G. brevicaudus*, *G. saxatilis*, and *G. ussuriensis*) belonging to the Viperidae family, as well as *Rhabdophis tigrinus* of the Colubridae family. Among the cases in which the species was identified, the *Gloydius* species accounted for 96.6% of the snakebite incidents in Korea [4].

Stroke has recently been recognized as a rare but severe complication of venomous snake envenomation and is often associated with high mortality rates or disabling neurological sequelae [5]. Several reviews of published cases—largely derived from non-Korean populations—have characterized stroke during the acute phase after snake envenomation. These reports indicate that most affected patients were young males without preexisting stroke risk factors, ischemic stroke predominated, and events typically occurred within 24 h of envenomation despite antivenom administration. Nearly half of the survivors experienced disabling neurological deficits [6,7]. Most reported cases were associated with Viperidae species.

Although previous evidence has focused primarily on stroke during the acute phase, an increased risk of stroke has also been suggested beyond the acute phase. A population-based study from Taiwan demonstrated an increased risk of hemorrhagic stroke during a 6-month to 3-year follow-up period after venomous snake envenomation among patients treated with antivenom targeting hemorrhagic toxic venom, in which coagulopathy is a characteristic clinical manifestation [8,9].

Korean data concerning stroke after snake envenomation are confined to three stroke cases during the acute phase. Two cases involved ischemic stroke—one occurring 3 h after envenomation with persistent neurological deficits and another fatal event occurring 3 days later [10,11]. One additional case involved intracerebral hemorrhage accompanied by subarachnoid hemorrhage approximately 6 h after envenomation, which resulted in death [12]. Although stroke during the acute phase after snake envenomation appears to be uncommon in Korea, these reports support the potential clinical severity of stroke after snake envenomation.

Given that the venom composition varies according to species, geography, and ontogeny, the clinical manifestations and cerebral sequelae of snake envenomation in Korea may not be directly generalizable from non-Korean data. Furthermore, the predominance of Viperidae snake envenomation and the frequent occurrence of venom-induced consumption coagulopathy in Korea provide a biologically plausible rationale for stroke development after snake envenomation [1]. Nevertheless, the long-term risk of stroke has not been systematically evaluated in Korea.

Therefore, we conducted a nationwide population-based cohort study to evaluate the long-term risk of stroke following snake envenomation in Korea.

## 2. Results

### 2.1. Clinical Characteristics of the Study Population

The baseline characteristics of the 764 case patients and 3056 matched controls are summarized in Table 1. The two groups were well balanced with respect to age, sex, and socioeconomic status due to matching. The prevalence of major comorbidities and the CCI score did not differ significantly between the case and control groups. Among the lifestyle factors, heavy alcohol consumption was more common in the case group than in the control group (29.5% vs. 24.5%, *p* < 0.01). The body mass index distribution also differed between groups, with obesity (BMI ≥ 25 kg/m^2^) being less common in the case group than in the control group (32.1% vs. 37.8%, *p* = 0.01), whereas the current smoking status and regular physical activity were comparable.

### 2.2. Main Outcome

During the 10-year follow-up period, participants were followed from the index date until stroke occurrence, death, or 31 December 2023, whichever occurred first. Total stroke occurred in 37 case patients and 137 controls, corresponding to crude incidence rates of 5.06 and 4.68 per 1000 person-years, respectively. In sequential multivariable Cox proportional hazards models, snake envenomation was significantly associated with an increased risk of total stroke in the model adjusted for age, sex, socioeconomic status, major comorbidities, CCI and lifestyle factors (Model 3 adjusted hazard ratio [aHR], 1.42; 95% confidence interval [CI], 1.01–1.99) (Table 2). When stroke subtypes were analyzed separately, snake envenomation was not associated with ischemic stroke after adjustment (Model 3 aHR, 1.05; 95% CI, 0.69–1.59), whereas it was significantly associated with an increased risk of hemorrhagic stroke (Model 3 aHR, 2.55; 95% CI, 1.12–5.80).

Diabetes mellitus remained independently associated with total stroke (aHR, 2.08; 95% CI, 1.49–2.90), ischemic stroke (aHR, 2.10; 95% CI, 1.40–3.15), and hemorrhagic stroke (aHR, 3.50; 95% CI, 1.65–7.42), whereas sex was not significantly associated with stroke outcomes after full adjustment (Table S1).

### 2.3. Severity-Stratified and Sex–Diabetes Interaction Analyses

In severity-stratified analyses, neither mild nor severe envenomation was significantly associated with total or ischemic stroke compared with the control group. However, severe envenomation was independently associated with an increased long-term risk of hemorrhagic stroke (aHR, 3.85; 95% CI, 1.17–12.68), whereas mild envenomation was not (Table 3).

Among the case group, subgroup analyses of patients stratified by sex and diabetes mellitus status showed that diabetes mellitus was significantly associated with increased risks of total (aHR, 2.43; 95% CI, 1.67–3.53), ischemic (aHR, 2.29; 95% CI, 1.49–3.51), and hemorrhagic strokes (aHR, 2.97; 95% CI, 1.37–6.43) in men (Table 4). However, no significant associations were observed in women. Interaction analyses demonstrated that the effect of diabetes mellitus on the stroke risk differed significantly by sex, with the strongest interaction observed for hemorrhagic stroke (*p* for interaction <0.01).

## 3. Discussion

In this nationwide population-based cohort study in Korea, snake envenomation was associated with an increased long-term risk of stroke, which was predominantly driven by hemorrhagic events rather than ischemic events. The increased long-term risk of hemorrhagic stroke after snake envenomation was particularly evident in men with diabetes mellitus as a baseline characteristic and in patients who experienced severe envenomation, as indicated by disseminated intravascular coagulation or the need for blood transfusion during the acute phase.

Globally, research on snake envenomation has focused primarily on stroke during the acute phase after envenomation, with limited evidence regarding the long-term stroke risk [6,7,13]. In Korea, reports have been restricted to three isolated stroke cases during the acute phase after snake envenomation [10,11,12]. This nationwide cohort study therefore provides longitudinal evidence from the Korean population suggesting an increased long-term risk of hemorrhagic stroke after snake envenomation. Similarly, a recent study from Taiwan reported an increased long-term risk of hemorrhagic stroke following viper envenomation [8].

We observed a clear difference between stroke occurring during the acute phase and stroke developing during long-term follow-up with respect to both the predominant subtype (ischemic stroke in the acute phase vs. hemorrhagic stroke during long-term follow-up) and the affected population (young patients without stroke risk factors in the acute phase vs. diabetic men during long-term follow-up) [6,7].

Several biological mechanisms may explain the association between stroke and snake envenomation. Ischemic stroke during the acute phase after snake envenomation has been proposed to involve venom-induced consumption coagulopathy-related thrombosis, venom-mediated vascular injury with secondary vasculitis, cardiotoxic embolism, and systemic hypotension or hypoperfusion [6,8]. In contrast, hemorrhagic stroke during the acute phase may be more closely related to endothelial cell injury, increased vascular permeability, thrombocytopenia, and coagulation abnormalities that lead to a bleeding-prone state. Snake venom metalloproteinases, which are abundant in the venom of *Gloydius brevicaudus*, a predominant venomous snake species in Korea, consume clotting factors, resulting in the subsequent depletion of fibrinogen and, ultimately, venom-induced consumption coagulopathy. Additionally, snake venom metalloproteinases degrade type IV collagen within the vascular basement membrane, resulting in compromised vascular integrity [14,15]. Because type IV collagen is essential to the mechanical stability of arterioles, capillaries, and postcapillary venules, its degradation may predispose affected vessels to hemorrhage. Despite these proposed mechanisms for stroke during the acute phase, the pathophysiology of stroke occurring months after snake envenomation remains largely unknown. The association between severe envenomation and subsequent hemorrhagic stroke may support the hypothesis that delayed hemorrhagic stroke could be related to venom-related systemic toxicity.

During the acute phase, clinical complications are largely driven by the circulating venom burden. Acute ischemic stroke may develop through transient venom-mediated prothrombotic states, even in patients without traditional vascular risk factors. As the toxin is cleared, this thrombotic risk likely declines. In contrast, hemorrhagic stroke occurring during long-term follow-up may reflect delayed vascular alterations beyond the acute phase. Such vascular alterations may involve endothelial injury caused by the metalloproteinases present in snake venom, even after apparent clinical recovery from envenomation manifestations such as venom-induced coagulopathy. When combined with diabetes-related vascular fragility and endothelial dysfunction, this residual vulnerability may contribute to an increased susceptibility to delayed hemorrhagic stroke beyond the acute phase, particularly in men. However, the exact mechanisms underlying hemorrhagic stroke occurring during long-term follow-up remain unclear.

Clinically, these findings indicate that the clinical consequences of snake envenomation may extend beyond the acute phase and include delayed hemorrhagic stroke in vulnerable populations. An awareness of this potential long-term risk may inform postacute monitoring and risk assessment strategies in vulnerable patients. Future studies should evaluate whether optimized acute management, including timely antivenom administration, may mitigate the long-term cerebrovascular risk.

Several limitations should be acknowledged. First, the specific venomous species responsible for envenomation could not be identified because no commercially available serologic assay is currently available for use in routine clinical practice in Korea. *Gloydius* species are reported to be responsible for most venomous snake bites in South Korea [16].

Second, stroke outcomes were identified using claims-based operational definitions without systematic neuroimaging confirmation, which may have introduced some misclassification. Although hospitalization (≥3 days) or repeated outpatient visits were used to improve diagnostic validity, some degree of outcome misclassification may still have remained. In addition, despite adjustment for multiple baseline covariates, residual confounding inherent to the observational study design cannot be excluded. Furthermore, mechanistic or biomarker data related to vascular dysfunction or endothelial injury were not available in this nationwide administrative database study.

Third, only patients with snake envenomation who received antivenom treatment were included to improve the specificity of clinically meaningful envenomation and reduce the inclusion of possible dry-bite cases. However, this approach may have excluded some milder envenomation cases not treated with antivenom. In addition, detailed information regarding the total antivenom dose, timing of administration, and treatment response was unavailable in the claims database. Therefore, we could not evaluate the potential influence of antivenom-related factors on subsequent stroke risk. Furthermore, the relatively small number of hemorrhagic stroke events may have limited the precision of certain subgroup analyses. Finally, because this study was conducted with data from the Korean healthcare system, the generalizability of the findings to other geographic regions or snake species may be limited.

## 4. Conclusions

These findings suggest a potential association between snake envenomation and an increased long-term risk of hemorrhagic stroke, particularly among men with diabetes and patients with severe envenomation. Further studies are needed to confirm these observations and clarify the underlying mechanisms.

## 5. Materials and Methods

### 5.1. Data Source and Ethics

This nationwide retrospective cohort study was conducted using the Korean National Health Insurance Service (NHIS) database, a government-administered healthcare system that covers nearly the entire Korean population [17].

The NHIS also provides biennial national health screening examinations and the results are linked to individual longitudinal healthcare records within the NHIS database. Since 2002, the NHIS database has accumulated nationwide longitudinal data on demographic characteristics and insurance claims, including International Classification of Diseases, Tenth Revision (ICD-10) diagnostic codes, treatment records, and prescription records, for more than 50 million individuals. Owing to its nationwide coverage and longitudinal structure, the NHIS database has been widely used in population-based epidemiological research.

In this study, healthcare utilization data were longitudinally tracked using encrypted anonymous identifiers. Because the study used retrospectively collected deidentified data, informed consent was waived. The study protocol was approved by the Institutional Review Board of Chonnam National University Hospital (IRB No. CNUH-EXP-2025-150).

### 5.2. Study Population

This nationwide cohort study used NHIS data from 1 January 2012 to 31 December 2023. Between 2012 and 2013, adult patients aged 18 years or older without prior diagnoses of toxic effect of snake venom (ICD-10 code T63.0), transient ischemic attack, vascular syndromes of brain, retinal artery occlusion, or cerebrovascular diseases were considered for inclusion. The case group consisted of adults (≥18 years) who were hospitalized with a diagnosis of T63.0 and received antivenom therapy during the index hospitalization between 1 January 2012 and 31 December 2013. For the case group, the index date was defined as the date of hospitalization for snake envenomation. Individuals without a record of the ICD-10 code T63.0 during the study period were eligible for the control group. Controls were exactly matched to patients with snake envenomation at a 4:1 ratio according to age, sex, and socioeconomic status. To minimize immortal time bias, matched controls were assigned the same index date as their corresponding case patients. Baseline balance after matching was evaluated using standardized mean differences, with values <0.1 considered indicative of adequate balance. Participants were followed from the index date until death, emigration, or the end of the study period.

### 5.3. Primary and Secondary Outcomes and Definitions

The primary outcome of this study was newly diagnosed stroke after snake envenomation. Total stroke was defined as any cerebrovascular event and was identified using the ICD-10 codes I60.x–I64.x. The secondary outcomes were hemorrhagic stroke (I60.x–I62.x) and ischemic stroke (I63.x). Stroke was identified on the basis of ICD-10 codes accompanied by hospitalization ≥3 days or ≥2 outpatient visits.

### 5.4. Data Collection

Information on demographic characteristics, socioeconomic status, comorbidities, and lifestyle factors was extracted from the NHIS database and linked national health screening data. Socioeconomic status was categorized into income-based quartiles according to health insurance premium levels. Major comorbidities, including diabetes mellitus (ICD-10 E10–E14), hypertension (I10–I15), dyslipidemia (E78), and chronic kidney disease (N18), were identified, and the Charlson Comorbidity Index (CCI) was calculated to quantify the overall comorbidity burden (Table S2). The CCI was originally developed in 1987 to estimate the 1-year mortality risk associated with coexisting medical conditions [18,19]. Because diabetes mellitus and chronic kidney disease were modeled separately in this study, a modified CCI excluding these components was used to avoid overlapping adjustment.

Lifestyle factors, including smoking status, alcohol consumption, physical activity level, and body mass index, were obtained from health screening records and included as potential confounders based on their established associations with stroke risk [20].

Among patients with snake envenomation, clinical severity was defined according to the presence of disseminated intravascular coagulation (ICD-10 D65) or the requirement for blood transfusion (Korean National Health Insurance procedure codes X2011, X2012, X2021, X2022, X2031, X2032, X2041, X2042, X2051, X2052, X2111, X2112, X2121, and X2122) within 2 days of the initial hospital visit. In a study of patients presenting within 24 h after snake envenomation, coagulation abnormalities consistent with venom-induced consumption coagulopathy, including international normalized ratio (INR), activated partial thromboplastin time (aPTT) and fibrinogen levels, reached their most extreme values within 16 h of hospital arrival. Based on this temporal profile, we defined severe envenomation using events occurring within 2 days of admission (e.g., DIC codes or transfusion), to capture early clinically significant coagulopathy while minimizing misclassification of later, unrelated events [21].

### 5.5. Statistical Analysis

After exact 1:4 matching by age, sex, and socioeconomic status, the baseline characteristics of the case and control groups were examined. Categorical variables are presented as frequencies with percentages and were analyzed using chi-square tests. Continuous variables are presented as the means with standard deviations or medians with interquartile ranges and were compared using Student’s *t* test or the Wilcoxon rank-sum test, as appropriate. A 1-year lag period was applied by excluding events occurring within the first year after the index date and initiating follow-up thereafter to evaluate the long-term stroke risk. Crude 10-year incidence rates (2014–2023) of stroke were calculated per 1000 person-years according to the snake envenomation status. Hazard ratios (HRs) and 95% confidence intervals (CIs) were estimated using multivariable Cox proportional hazards regression models with baseline covariates. The proportional hazards assumption was assessed using Schoenfeld residuals and visual inspection of log-minus-log survival curves, and no significant violations were identified. Missing covariate data were handled using Multiple Imputation by Chained Equations (MICE). Five imputed datasets were generated, and pooled estimates were used for the fully adjusted Cox proportional hazards regression analyses. The following sequential adjustment models were constructed: Model 1 included the matching variables (age, sex, and socioeconomic status); Model 2 was additionally adjusted for major baseline comorbidities, including the CCI; and Model 3 was further adjusted for lifestyle factors (smoking status, alcohol consumption, physical activity level, and body mass index). Fully adjusted estimates from Model 3 were considered the primary results. Multicollinearity among covariates was assessed to confirm the model’s validity.

The severity-stratified analyses were performed by classifying the case group into mild and severe envenomation according to the presence of disseminated intravascular coagulation or the requirement for blood transfusion within 2 days, with the matched control group used as the reference.

Additional subgroup and interaction analyses according to sex and diabetes mellitus status were conducted in the case group using Cox proportional hazards regression models.

Statistical analyses were conducted with SAS software (version 9.4; SAS Institute, Cary, NC, USA). Statistical significance was defined as a two-tailed *p* value less than 0.05.

## Figures and Tables

**Table 1:** Baseline characteristics of the study population according to snake envenomation status.

Variables	All	Snake Envenomation		*p* Value
		Yes	No	
Number of patients	3820 (100.0)	764 (100.0)	3056 (100.0)	
Age (year)				>0.99
18–39	950 (24.9)	190 (24.9)	760 (24.9)	
40–64	2270 (59.4)	454 (59.4)	1816 (59.4)	
≥65	600 (15.7)	120 (15.7)	480 (15.7)	
Male	2710 (70.9)	542 (70.9)	2168 (70.9)	>0.99
Socioeconomic status				>0.99
Low	1050 (27.5)	210 (27.5)	840 (27.5)	
Middle	1300 (34.0)	260 (34.0)	1040 (34.0)	
High	1470 (38.5)	294 (38.5)	1176 (38.5)	
Comorbidity				
Hypertension	690 (18.1)	145 (19.0)	545 (17.8)	0.49
Diabetes mellitus	529 (13.8)	114 (14.9)	415 (13.6)	0.37
Dyslipidemia	547 (14.3)	115 (15.1)	432 (14.1)	0.56
Coronary heart disease	56 (1.5)	24 (3.1)	104 (3.4)	0.81
Chronic kidney disease	154 (4.0)	35 (4.6)	119 (3.9)	0.45
CCI Score (Mean ± SD)	1.4 ± 1.0	1.4 ± 1.1	1.4 ± 1.0	0.75
Life style factors				
Body mass index, kg/m^2^				0.01
<18.5 (Underweight)	106 (2.8)	21 (2.7)	85 (2.8)	
18.5–22.9 (Normal)	1341 (35.1)	302 (39.5)	1039 (34.0)	
23.0–24.9 (Overweight)	974 (25.5)	196 (25.7)	778 (25.5)	
≥25 (Obese)	1399 (36.6)	245 (32.1)	1154 (37.8)	
Current Smoker	722 (18.9)	131 (17.1)	591 (19.3)	0.18
Heavy alcohol drinker	975 (25.5)	225 (29.5)	750 (24.5)	<0.01
Regular physical activity	1436 (37.6)	275 (36.0)	1161 (38.0)	0.33

Body mass index was categorized using the Asia–Pacific criteria as follows: underweight (<18.5 kg/m^2^), normal weight (18.5–22.9 kg/m^2^), overweight (23.0–24.9 kg/m^2^), and obesity (≥25.0 kg/m^2^). Heavy alcohol intake referred to alcohol consumption of at least 30 g/day. Regular physical activity was considered present when participants reported moderate exercise for ≥5 days/week or vigorous exercise for ≥3 days/week. CCI, Charlson Comorbidity Index. The values are presented as numbers (%), unless indicated otherwise.

**Table 2:** Incidence rates and multivariable-adjusted hazard ratios for stroke according to the snake envenomation status.

Stroke			Patients (*n*)	Stroke Events (*n*)	Person-Years	Incidence Rate per 1000 PY	Model 1	Model 2	Model 3
							aHR (95% CI)	aHR (95% CI)	aHR (95% CI)
Total stroke	Snake envenomation	No	3056	137	29,291.20	4.68	1.00 (reference)	1.00 (reference)	1.00 (reference)
		Yes	764	37	7309.60	5.06	1.08 (0.75–1.56)	1.21 (0.85–1.73)	1.42 (1.01–1.99)
Ischemic stroke	Snake envenomation	No	3056	110	29,291.20	3.76	1.00 (reference)	1.00 (reference)	1.00 (reference)
		Yes	764	29	7309.60	3.97	1.06 (0.70–1.59)	0.98 (0.65–1.48)	1.05 (0.69–1.59)
Hemorrhagic stroke	Snake envenomation	No	3056	27	29,291.20	0.92	1.00 (reference)	1.00 (reference)	1.00 (reference)
		Yes	764	8	7309.60	1.09	1.19 (0.54–2.61)	1.95 (0.88–4.32)	2.55 (1.12–5.80)

The adjustment strategy was sequentially constructed across the three models. The first model included age, sex, and socioeconomic status. The second model additionally incorporated major baseline comorbidities, including hypertension, diabetes mellitus, dyslipidemia, chronic kidney disease, and the Charlson Comorbidity Index. The fully adjusted third model further accounted for lifestyle-related variables, including body mass index, smoking status, alcohol intake, and physical activity. PY, person-years; aHR, adjusted hazard ratio; CI, confidence interval.

**Table 3:** Long-term stroke risk according to the severity of snake envenomation.

Outcome	Severity Subgroup	Patients (*n*)	Stroke Events (*n*)	Incidence Rate (per 1000 PY)	Adjusted HR (95% CI)
Total Stroke	Control (Ref)	3056	137	4.82	1.00 (Reference)
	Mild snake envenomation	670	30	4.82	1.00 (0.67–1.48)
	Severe snake envenomation	94	7	8.13	1.60 (0.75–3.42)
Ischemic Stroke	Control (Ref)	3056	110	3.87	1.00 (Reference)
	Mild snake envenomation	670	25	4.01	1.06 (0.68–1.63)
	Severe snake envenomation	94	4	4.64	1.20 (0.44–3.25)
Hemorrhagic Stroke	Control (Ref)	3056	27	0.95	1.00 (Reference)
	Mild snake envenomation	670	5	0.8	0.80 (0.31–2.08)
	Severe snake envenomation	94	3	3.48	3.85 (1.17–12.68)

Adjusted hazard ratios (aHRs) were estimated using Cox proportional hazards models adjusted for age, socioeconomic status, hypertension, diabetes mellitus, dyslipidemia, chronic kidney disease, the Charlson Comorbidity Index, and lifestyle-related variables, including body mass index, smoking status, alcohol intake, and physical activity. PY, person-years; aHR, adjusted hazard ratio; CI, confidence interval.

**Table 4:** Sex-stratified association between diabetes mellitus and stroke among patients with snake envenomation.

Outcome	Sex	Diabetes Mellitus	Patients (*n*)	Stroke Events (*n*)	Incidence Rate (per 1000 PY)	Adjusted HR (95% CI)	*p* for Interaction
Total Stroke							
	Male	No (Ref)	2301	88	3.99	1	0.042
		Yes	409	40	10.22	2.43 (1.67–3.53)	
	Female	No (Ref)	990	41	4.31	1	
		Yes	120	5	4.34	0.96 (0.38–2.42)	
Ischemic Stroke							
	Male	No (Ref)	2301	70	3.18	1	0.035
		Yes	409	30	7.66	2.29 (1.49–3.51)	
	Female	No (Ref)	990	35	3.68	1	
		Yes	120	4	3.47	0.90 (0.32–2.52)	
Hemorrhagic Stroke							
	Male	No (Ref)	2301	18	0.82	1	<0.01
		Yes	409	10	2.55	2.97 (1.37–6.43)	
	Female	No (Ref)	990	6	0.63	1	
		Yes	120	1	0.87	1.30 (0.16–10.82)	

Adjusted hazard ratios (aHRs) were estimated using Cox proportional hazards models adjusted for age, socioeconomic status, hypertension, dyslipidemia, chronic kidney disease, the Charlson Comorbidity Index, and lifestyle related variables. *p* for interaction represents the statistical significance of the interaction between sex and diabetes mellitus for stroke outcomes. PY, person-years; HR, hazard ratio; CI, confidence interval.

## Data Availability

The data used in this study were obtained from the Korean National Health Insurance Service and are subject to institutional access restrictions. Researchers seeking access to the dataset are required to obtain approval directly from the Korean National Health Insurance Service through appropriate application procedures.

## Supplementary Materials

The following supporting information can be downloaded at: https://www.mdpi.com/article/10.3390/toxins18060265/s1, Table S1: Incidence rates and multivariable-adjusted hazard ratios for stroke in patients stratified according to the diabetes mellitus status and sex.; Table S2: Operational definitions and coding criteria for major variables.
