# Supplementary material for: Long-Term Risk of Stroke After Snake Envenomation: A Nationwide Population-Based Cohort Study in Korea
Source: Toxins (Basel). 2026 Jun 12;18(6):265. doi: 10.3390/toxins18060265 (PMC13307708; doi:10.3390/toxins18060265)
Supplement: Supplementary file 1 [file toxins-18-00265-s001.zip › toxins-4255039-supplementary.pdf]

Supplementary Materials: Long-Term Risk of Stroke After Snake Envenomation: A Nationwide Population-Based Cohort Study in Korea

Table S1. Incidence rates and multivariable-adjusted hazard ratios for stroke in patients stratified according to the diabetes mellitus status and sex.

Stroke			Patients (n)	Stroke events (n)	Person-years	Incidence rate per 1000 PY	Model 1	Model 2	Model 3
							aHR (95% CI)	aHR (95% CI)	aHR (95% CI)
Total stroke	Sex	Female	1,110	46	10,657.20	4.32	1.00 (reference)	1.00 (reference)	1.00 (reference)
		Male	2,710	128	25943.6	4.93	1.14 (0.82-1.60)	1.18 (0.85-1.63)	1.25 (0.89-1.76)
	Diabetes Mellitus	No	3,291	129	31,632.60	4.08	1.00 (reference)	1.00 (reference)	1.00 (reference)
		Yes	529	45	4,968.20	9.06	2.22 (1.58-3.12)	2.15 (1.55-2.98)	2.08 (1.49-2.90)
Ischemic stroke	Sex	Female	1110	39	10,657.20	3.66	1.00 (reference)	1.00 (reference)	1.00 (reference)
		Male	2710	100	25,943.60	3.85	1.05 (0.73-1.52)	1.05 (0.73-1.52)	1.10 (0.75-1.60)
	Diabetes Mellitus	No	3291	105	31,632.60	3.32	1.00 (reference)	1.00 (reference)	1.00 (reference)
		Yes	529	34	4,968.20	6.84	2.06 (1.40-3.04)	2.15 (1.45-3.18)	2.10 (1.40-3.15)
Hemorrhagic stroke	Sex	Female	1110	7	10,657.20	0.66	1.00 (reference)	1.00 (reference)	1.00 (reference)
		Male	2710	28	25,943.60	1.08	1.64 (0.72-3.76)	1.75 (0.75-4.10)	1.85 (0.80-4.25)
	Diabetes Mellitus	No	3291	24	31,632.60	0.76	1.00 (reference)	1.00 (reference)	1.00 (reference)
		Yes	529	11	4,968.20	2.21	2.92 (1.43-5.96)	3.20 (1.55-6.60)	3.50 (1.65-7.42)

Table S2. Operational definitions and coding criteria for major variables.

Variables	Operational definition	Codes/criteria
Snake envenomation	Hospitalization with toxic effect of snake venom and antivenom treatment	ICD-10 codes T63.0
Total stroke	Hospitalization ≥3 days or ≥2 outpatient visits with stroke diagnosis	ICD-10 codes I60–I64
Hemorrhagic stroke	Hospitalization ≥3 days or ≥2 outpatient visits with hemorrhagic stroke diagnosis	ICD-10 codes I60–I62
Ischemic stroke	Hospitalization ≥3 days or ≥2 outpatient visits with ischemic stroke diagnosis	ICD-10 codes I63
Hypertension	Diagnosis of hypertension	ICD-10 codes I10–I15
Diabetes mellitus	Diagnosis of diabetes mellitus	ICD-10 codes E10–E14
Dyslipidemia	Diagnosis of dyslipidemia	ICD-10 codes E78
Chronic kidney disease	Diagnosis of chronic kidney disease	ICD-10 codes N18
Disseminated intravascular coagulation	Diagnosis of DIC	ICD-10 codes D65
Blood transfusion	Blood transfusion during hospitalization	Korean NHIS procedure codes X2011, X2012, X2021, X2022, X2031, X2032, X2041, X2042, X2051, X2052, X2111, X2112, X2121, and X2122
Smoking status	Self-reported current smoking status in the index-year NHIS health examination	National health screening questionnaire
Heavy alcohol consumption	Alcohol consumption ≥30 g/day	National health screening questionnaire
Regular physical activity	Moderate-intensity physical activity ≥5 times/week or vigorous activity ≥3 times/week	National health screening questionnaire
Body mass index	Calculated as weight (kg)/height² (m²)	Categorized as <18.5, 18.5–22.9, 23.0–24.9, or ≥25.0 kg/m²
